# Protocol: The Effects of Communication Strategies on Upcycled Food Acceptance: A Systematic Review and Meta‐Analysis

**DOI:** 10.1002/cl2.70075

**Published:** 2025-11-14

**Authors:** Shuai Ma, Zhihong Xu, Peng Lu, Jean Parrella, Ashlynn Kogut

**Affiliations:** ^1^ Texas A&M University College Station Texas USA; ^2^ University of Georgia Athens Georgia USA; ^3^ Virginia Tech Blacksburg Virginia USA

## Abstract

This is the protocol for a Campbell systematic review. The objectives are as follows. Our review will exclusively emphasize quantitative evidence from experimental studies, identifying the important factors and providing comprehensive and in‐depth recommendations, with a primary focus on identifying the effective communication and marketing strategies that have been evaluated. The findings of the study focusing on consumers' acceptance will provide valuable insight for policymakers to combat the food waste issue. The research questions are as follows: RQ1: What are the key factors influencing consumer acceptance in experimental studies on upcycled foods? RQ2: What communication and marketing strategies have been used to increase consumer acceptance in experimental studies on upcycled foods?

## 1 Background

### 1.1 Food Waste Issue

Global food waste, particularly pre‐consumer waste, poses a significant challenge that demands innovative solutions and concerted efforts to reduce its negative impact on both the environment and food security. Efforts have been made at the policy level to combat food waste, with goals set by organizations like the U.S. Department of Agriculture (USDA) in 2013 and the United Nations (UN) in 2015. In 2013, a collaborative initiative known as the U.S. Food Waste Challenge was launched by the USDA and the Environmental Protection Agency (EPA). This initiative sought to transform how various stakeholders along the food supply chain managed food and food waste (U.S. Department of Agriculture 2013). Both the USDA and the UN have established targets to reduce food waste by 2030. For instance, the USDA aims to achieve a 50% reduction in food waste by 2030 by enhancing the efficiency of the food supply chain (U.S. Department of Agriculture 2015). Furthermore, there are policies in place that specifically promote upcycled food as a solution to the food waste problem. Both government and nonprofit entities, such as the UN and Rethink Food Waste Through Economics and Data (ReFED), have recognized the potential benefits of upcycled food as a solution to reduce waste in food production and supply chains (UN 2017, 22; ReFED 2020).

Recently, researchers, policymakers, and food industry stakeholders have paid attention to upcycled food due to its potential to reduce food waste (Aschemann‐Witzel et al. 2023; Goodman‐Smith et al. 2021). Upcycling food involves converting food waste or by‐products into valuable new products (Bhatt et al. 2018; Moshtaghian et al. 2021). Several definitions of upcycled foods have been proposed. For example, in 2020, Spratt et al. defined it as follows: “Upcycled ingredients and food products elevate food that would otherwise be wasted to higher uses and have tangible benefits to the environment and society” (p. 491). In 2022, the Upcycled Food Association, the first nonprofit organization dedicated to improving the upcycled food supply chain, proposed that “upcycled foods use ingredients that would otherwise not be destined for human consumption, are procured and produced using verifiable supply chains, and have a positive impact on the environment” (Upcycled Food Association 2022). The most recent definition was introduced by Aschemann‐Witzel et al. (2023); they recommended three characteristics that upcycled food should have: “(1) a product consisting of or containing materials that would otherwise be waste, (2) turned into a food product for human consumption, and (3) a process that involves an increase in value” (p. 133). In this study, we use the definition proposed by UFA in 2022 and Aschemann‐Witzel et al. (2023), which combines components like food waste, human consumption, value‐added product, and positive impact on the environment. Opting for upcycled food items can greatly aid environmental conservation efforts and diminish food waste.

### 1.2 Factors Affect Consumers' Acceptance of Upcycled Food

Studies suggest that consumer choices can significantly impact the reduction of food waste at the beginning of the supply chain, particularly through the selection of certain food items (Aschemann‐Witzel et al. 2022). Interest in this area of research has grown, particularly after Bhatt et al. (2018) publication, with various studies investigating the influence of product information on consumer attitudes and their willingness to invest in upcycled foods. Consumer acceptance refers to the overall attitude, readiness/willingness to pay, and their acceptance choice/decision to accept upcycled food. The understanding of consumer acceptance and their willingness to pay for upcycled food remains limited (Aschemann‐Witzel et al. 2022; Aschemann‐Witzel and Peschel 2019; Aschemann‐Witzel and Stangherlin 2021; Grasso and Asioli 2020). Some studies have indicated that consumers are hesitant to pay for upcycled foods because they are unfamiliar (Bhatt et al. 2020). Therefore, it is crucial to investigate the most important factors that deter consumer behavior, and the effect of the significant factors based on evidence from experimental studies.

### 1.3 Informational Intervention

The communication strategies employed by UFA emphasized benefits related to sustainability, health, and the economy, with a particular focus on highlighting environmental information for upcycled foods (Ma et al. 2024). It is important to be aware of the sustainability and potential profitability of upcycled food. The success of marketing upcycled food is closely tied to how consumers perceive and accept these products.

### 1.4 Research Gap

Until now, five reviews have been published to synthesize existing literature. In 2021, Moshtaghian et al. conducted a literature review that identified factors influencing consumer acceptance of upcycled foods. These factors were sociodemographic characteristics, attitudes toward food waste management, sustainability, and the environment, as well as food quality characteristics (Moshtaghian et al. 2021). In addition, Aschemann‐Witzel and Stangherlin (2021) conducted a systematic review, which found that individual factors (e.g., gender, age), contextual factors (e.g., communication), and product‐related factors (e.g., food type, production methods) influence consumers' acceptance of waste‐to‐value foods. Later, Aschemann‐Witzel et al. (2023) conducted a literature review to present a comprehensive definition of upcycled foods to shape consumers' perceptions. In the same year, Ye (2023) conducted a literature review focusing on marketing perspectives and insights to increase consumer acceptance of upcycled foods. In 2024, Lu et al. conducted a scoping review to synthesize factors influencing consumers' acceptance of upcycled foods. They identified three categories influencing consumers' acceptance: sociodemographic characteristics, psychographic characteristics, and product characteristics.

There are limited studies on the overall impact of upcycled food experiments or effective marketing approaches (Asioli and Grasso 2021). Nevertheless, there is a scarcity of comprehensive reviews synthesizing experimental studies to assess the impacts and formulate strategies and recommendations for upcycled food marketing. The current body of research on upcycled food predominantly concentrates on technical aspects, with limited attention given to societal and economic implications or the consumer perspective (Aschemann‐Witzel and Stangherlin 2021). Aschemann‐Witzel and Stangherlin (2021) categorize acceptance into individual, contextual, and product‐related factors, while Ye (2023) summarizes acceptance in terms of consumer identities, consumption occasions, and word of mouth. None of the previous reviews has extensively explored the quantitative impact using empirical evidence from experimental studies.

## 2 Objectives

Our review will exclusively emphasize quantitative evidence from experimental studies, identifying the important factors and providing comprehensive and in‐depth recommendations, with a primary focus on identifying the effective communication and marketing strategies that have been evaluated. The findings of the study focusing on consumers' acceptance will provide valuable insight for policymakers to combat the food waste issue. The research questions are as follows:

RQ1: What are the key factors influencing consumer acceptance in experimental studies on upcycled foods?

RQ2: What communication and marketing strategies have been used to increase consumer acceptance in experimental studies on upcycled foods?

## 3 Methods

### 3.1 Criteria for Considering Studies for This Review

#### 3.1.1 Types of Studies

First, we will incorporate research articles published in peer‐reviewed journals, gray literature including dissertations/theses, conference proceedings, the websites of some organizations (e.g., UFA), and Google Scholar. The studies must be written in English. We will include the studies in a list of non‐English language publications in an annex. Second, we will exclusively consider quantitative studies, as our goal is to identify and consolidate factors that are connected with or influence the key outcome variable. Third, the included studies will be required to be experimental in nature.

As for our criteria for exclusion, we will exclude review studies like scoping reviews, literature reviews, and systematic reviews. In addition, we will exclude non‐experimental studies. Studies only examining the associations between factors and willingness to pay will be excluded.

#### 3.1.2 Types of Participants

The participants in the selected studies need to be 18 years of age or older to ensure they represented food purchasers and contributed to the consumer perspective.

#### 3.1.3 Types of Interventions

We anticipate the intervention to be an informational/message intervention. For the intervention, product‐related factor benefit information is of great interest. The information framing strategy can include the use of health benefit information versus no use or the use of sustainability benefit information versus no use. We can examine the overall effect of the information framing intervention for the review.

#### 3.1.4 Types of Outcome Measures

Each study must primarily assess consumer acceptance of upcycled foods, such as measuring purchase intention and willingness to pay.

#### 3.1.5 Duration of Follow‐Up

Duration of follow‐up will not be examined/documented.

#### 3.1.6 Types of Settings

Research investigating consumer acceptance of imperfect, ugly, suboptimal, abnormally shaped, or sustainable food items, because these fall into the category of whole foods and not foods made from upcycled ingredients. We will exclude studies that exclusively focus on aspects like food technology, household food waste behavior, and sensory evaluation of food products. We only serve upcycled food.

### 3.2 Search Methods for Identification of Studies

#### 3.2.1 Electronic Searches

We will conduct searches across six databases: CAB Abstracts (Ovid), Food Science and Technology Abstracts (EBSCO), Agricola (EBSCO), Business Source Ultimate (EBSCO), the Web of Science Core Collection, and Communication Source (EBSCO), covering areas such as agriculture, food science, applied life sciences, and business. The search strategy will be initially formulated in CAB Abstracts and then adapted and applied to the other four databases. Our search strategy will be on two key concepts: upcycled foods and their acceptance by consumers. Some of the keywords for upcycled foods are “agricultural byproducts,” “agroindustrial byproducts,” “meat byproducts,” “milk byproducts,” “seafood byproducts,” “upcycled food,” “upcycled ingredient,” “upcycled waste,” “value‐added food,” “circular food,” “rescue‐based food,” “waste‐to‐value product,” “waste‐to‐value food,” “food byproduct,” and “food waste.” Some of the keywords for acceptance by consumers are “consumer,” “consumer attitudes,” “consumer behavior,” “consumer preferences,” “consumer satisfaction,” “consumer belief,” “consumer perception,” “consumer willingness to pay,” and “consumer willingness to buy.” For each concept, we will pinpoint relevant terms within each database and combine these terms with associated keywords in titles and abstracts using the “or” operator. We will impose no date limitations on our searches, which were last carried out on February 27, 2024, for the initial search. The results from each database will be uploaded to Covidence, a web‐based tool for systematic review management, to remove duplicates and facilitate screening.

#### 3.2.2 Searching Other Resources

We will search the ProQuest Dissertations and Theses. We will also search Other gray literature sources like conference proceedings, websites of some organizations (e.g., UFA), and Google Scholar.

### 3.3 Data Collection and Analysis

#### 3.3.1 Description of Methods Used in Primary Research

We are particularly interested in the results of the included experimental studies, such as identifying factors that influence consumer acceptance and marketing/communication strategies used. We will conduct a systematic literature review to identify and summarize research about upcycled food factors influencing consumer acceptance, and the effects of those marketing strategies associated with the factors. The frequency with which a factor appears in the included studies will be identified as an important factor and guide the qualitative synthesis. We will use a meta‐analysis approach to quantify the magnitude of the most important factor's effect. A detailed synthesis of the recommended marketing strategies will provide guidance for practice and research.

**Figure 1:**
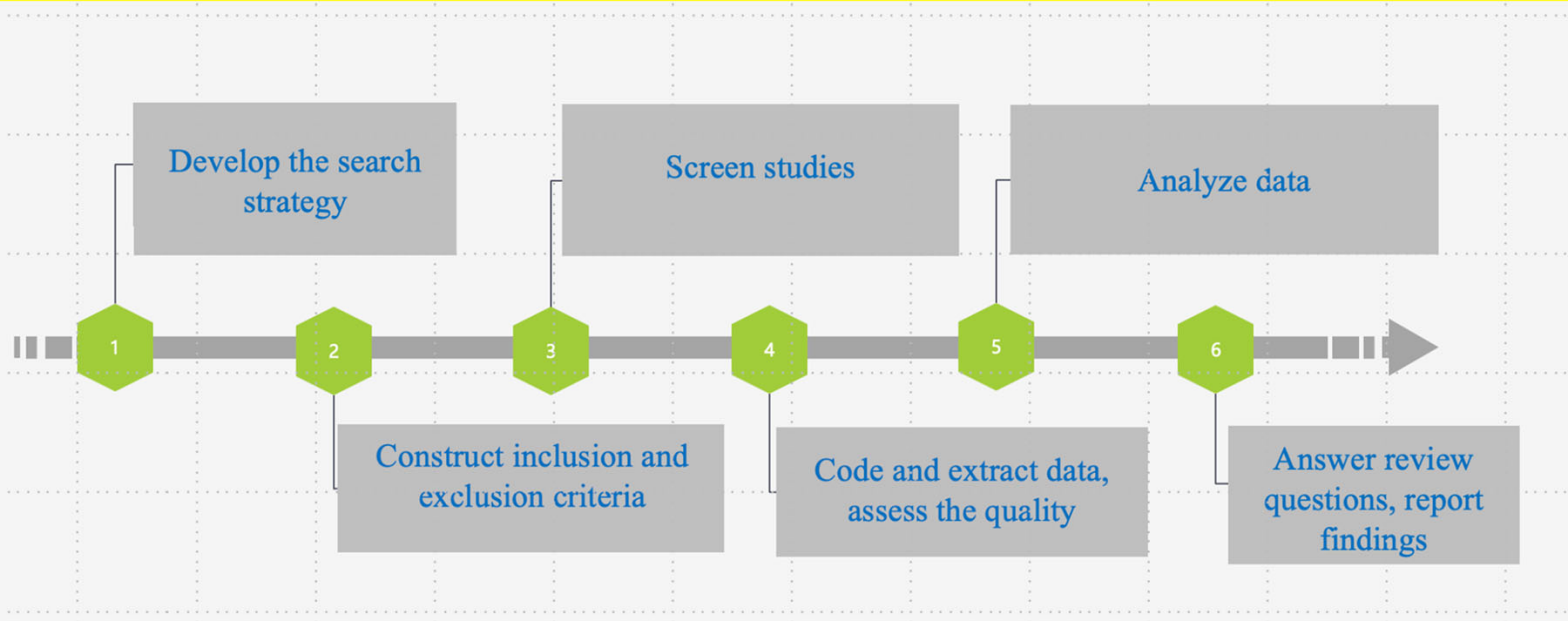
Systematic review procedure.

The process of systematic reviews includes developing research questions, the selection criteria, the search strategy, screening studies using selection criteria, coding studies, assessing the quality of studies, synthesizing the results based on the review questions, and reporting the findings (Newman and Gough 2020). Adopted from this process, a graphic representation of the procedure employed in this study is presented in Figure 1.

#### 3.3.2 Selection of Studies

Two authors will independently conduct title and abstract screening and full‐text screening. When screening the studies, we will adhere to pre‐established inclusion and exclusion criteria. The included studies agreed upon by two authors will go to the next phase of full‐text screening. The assessment of risk of bias will be conducted for each individual study. Two independent reviewers will apply these tools systematically, with discrepancies resolved by a third reviewer if necessary. When there is disagreement between two authors, a discussion will take place to resolve the conflict according to the inclusion and exclusion criteria.

#### 3.3.3 Data Extraction and Management

Two independent reviewers will code the coding sheet separately, with discrepancies resolved by a third reviewer if necessary. When there is disagreement between two coders, a discussion will take place to resolve the conflict. We will use Microsoft Excel as our tool for documenting coding and extraction. The detailed coding schema is as follows:

Study design: Randomized Controlled Trial (RCT) refers to where participants will be randomly assigned to conditions. Quasi‐experimental designs (e.g., non‐random group assignment, absence of a formal control group) will also be coded. Other emerging experimental designs will be coded as reported.

Factors: The term “factors” relates to the broad categories of determinants that influence consumer acceptance. These are segmented into sociodemographic factors, psychographic factors, and product‐related factors (Lu et al. 2024).

Type of Product Information: Product information includes product information related to environmental/sustainability benefits, health/nutrition benefits, and economic benefits/circular economy.

Communication Strategy: This concept refers to the marketing and communication strategies explored or recommended within the study. It is coded as presented in the study using a content analysis approach.

#### 3.3.4 Assessment of Risk of Bias in Included Studies

Risk of bias: We will conduct a quality assessment of the included studies. We will use the quality assessment instrument based on Petticrew and Roberts' framework for appraising a survey (Petticrew and Roberts 2006) and the Joanna Briggs Institute's Critical Appraisal Checklist for Analytical Cross‐Sectional Studies (Moola et al. 2017). Three categories will be used to appraise the risk of bias: high, moderate, and low. Studies reached a yes score of 49% will be identified as high, studies reached yes scores of 50% to 69% will be labeled as moderate, and studies reached yes scores of more than 74% will be identified as low (Baker et al. 2022).

#### 3.3.5 Measures of Treatment Effect

For RQ2, we will first evaluate the study design and calculate the effect size, representing the magnitude of the selected factor's impact on consumer acceptance. This analysis will be limited to studies employing an experimental study design (e.g., RCT or quasi‐experimental designs) and statistical methods. The overall effect (the summary effect size) will be calculated using the statistical software R. For the study design, the included studies will not be traditional intervention studies in education; instead, they are examining preference data (willingness to pay/intention to pay/attitude) through conducting an experiment. So, the experiment design type included can be RCT, where participants were randomly assigned to conditions, or a quasi‐experimental design, or another experimental design. The outcome (willingness to pay/likelihood to buy) is measured by a survey, which can provide a mean. The common measure is a Likert scale survey. The choice of effect measure will be the standardized mean difference. Mean, SD of each group will be used to calculate effect size. We will use a random‐effects model in the meta‐analysis to account for variation both within and between studies for the overall effect. This model assumes that the true effect size may vary across studies, and it provides a more conservative estimate than a fixed‐effects model.

#### 3.3.6 Unit of Analysis Issues

We do not anticipate the unit of analysis issue, as most studies do not involve repeated measures, clusters, or a crossover design.

#### 3.3.7 Criteria for Determination of Independent Findings

We will assess study independence by identifying multiple results/outcomes from the same study and including only the most relevant data or the most representative outcome for each article in the meta‐analysis.

#### 3.3.8 Dealing With Missing Data

If there are missing outcome data for the meta‐analysis, we will contact the author team to request clarification or additional data when necessary.

#### 3.3.9 Assessment of Heterogeneity

To test Heterogeneity, a statistical test will be employed (Cochran's *Q* Test). A significant *Q* statistic (*p*‐value < 0.05) suggests the presence of heterogeneity. If substantial heterogeneity is detected, we will further explore its sources and determine whether a meta‐analysis remains appropriate. Should it not be feasible, we will focus on a qualitative synthesis to ensure meaningful insights are still derived from the data, highlighting key patterns and effective strategies.

#### 3.3.10 Assessment of Reporting Biases

To assess reporting bias, including publication bias, we will visually inspect funnel plots for asymmetry in the meta‐analysis. Additionally, we will apply statistical tests, such as Egger's, to detect potential small‐study effects.

#### 3.3.11 Data Synthesis

For RQ1, we will first use a systematic review approach to identify the factors through content analysis. Based on the literature, factors can be divided into psychographic factors, sociodemographic factors, and product‐related factors. We will synthesize the factors emerging for the systematic review based on this framework. The results from the systematic review of the factors will be used to determine the focus/factors to analyze the effect using a meta‐analysis approach. Studies investigating the strategies related to product‐related factors (e.g., health benefit information, sustainability benefit information, economic benefit information) would be the focus. For RQ2, the meta‐analysis will investigate the effectiveness of the information framing strategy. We will use a random‐effects model in the meta‐analysis. We will investigate the information framing strategy (e.g., the use of health benefit information vs. no use or the use of sustainability benefit information vs. no use) to investigate the overall effect. For the intervention, product‐related factor/information is of great interest.

If a meta‐analysis is not feasible due to insufficient information provided to conduct a meta‐analysis or heterogeneity, we will only employ a systematic review/narrative synthesis of the factors and effective strategies. We will lastly identify the marketing or communication strategies used through content analysis. For the systematic review, different strategies will be summarized/synthesized/compared, reporting the information of positive, negative, or non‐significant effects.

#### 3.3.12 Subgroup Analysis and Investigation of Heterogeneity

We are primarily interested in investigating the overall effect. However, if sufficient studies related to information framing can be identified in each subgroup (health benefit information, economic benefit information, environmental benefit information), moderator analysis/subgroup analysis will be conducted. In addition, study design will be coded (RCT, quasi‐experimental design, etc.), and risk of bias (high, moderate, low) will also be used as a moderator if sufficient studies are identified under each subgroup. We will use a random effect model to generate the overall effect.

#### 3.3.13 Sensitivity Analysis

We will conduct a sensitivity analysis to assess the robustness and reliability of the overall findings.

#### 3.3.14 Treatment of Qualitative Research

We only included empirical studies that focus on quantitative methods, so that the qualitative research articles will be excluded.

#### 3.3.15 Summary of Findings and Assessment of the Certainty of the Evidence

The results will be presented in three parts. First, results from the systematic review will be reported, including identification of the factors influencing consumer acceptance. Moreover, results from the meta‐analysis will be reported, including the summary effect size of the important factor. If sufficient studies are identified for moderators (information type, risk of bias, and study design) for each group, the moderator analysis results will be provided. Lastly, we will report the marketing and communication strategies employed and discuss the study's implications and provide recommendations for increasing consumers' acceptance.

## Author Contributions

The review team includes members with diverse and complementary expertise to ensure the quality and rigor of the review. Conceptualization (Content Expertise): Peng Lu, Jean A. Parrella; Methodology (Systematic Review Methods): Zhihong Xu; Formal Analysis (Statistical Expertise): Shuai Ma; Data Curation (Information Retrieval Expertise): Ashlynn Kogut.

## Conflicts of Interest

The authors declare no conflicts of interest.

## Preliminary Timeframe

Date to submit a draft review: October 1, 2025.

## Plans for Updating This Review

At this time, we do not have plans to update this review.

## Peer Review

The peer review history for this article is available at https://www.webofscience.com/api/gateway/wos/peer-review/10.1002/cl2.70075.

## Supporting information

Appendix.

## Data Availability

Data will be available. Data coding sheet and analytic codes will be submitted as supporting materials. The data that support the findings of this study are available in the supporting material of this article.
