# Supplementary material for: Protocol: The Effects of Communication Strategies on Upcycled Food Acceptance: A Systematic Review and Meta‐Analysis
Source: Campbell Syst Rev. 2025 Nov 14;21(4):e70075. doi: 10.1002/cl2.70075 (PMC12617439; doi:10.1002/cl2.70075)
Supplement: Supplementary file 1 — Appendix. [file CL2-21-e70075-s001.docx]

# **Appendices**

#### Database Search strategies.

CAB Abstracts (Ovid)

1 food wastes/

2 agricultural byproducts/

3 agroindustrial byproducts/

4 meat byproducts/

5 milk byproducts/

6 seafood byproducts/

7 (upcycl* adj3 food*).ab,ti.

8 (upcycl* adj3 ingredient*).ab,ti.

9 (upcycle* adj3 waste*).ab,ti.

10 (value-added adj1 food).ab,ti.

11 (value added adj1 food).ab,ti.

12 (circular adj1 food*).ab,ti.

13 (Rescue-based adj1 food*).ab,ti.

14 (Rescue based adj1 food*).ab,ti.

15 (waste-to-value adj1 product*).ab,ti.

16 (waste-to-value adj1 food*).ab,ti.

17 (food* adj3 by-product*).ab,ti.

18 (food* adj3 "by product*").ab,ti.

19 (food* adj3 byproduct*).ab,ti.

20 food* waste*.ab,ti.

21 or/1-20

22 consumers/

23 consumer attitudes/

24 consumer behaviour/

25 consumer preferences/

26 consumer satisfaction/

27 "consumer*".ab,ti.

28 "accept*".ab,ti.

29 "adopt*".ab,ti.

30 "prefer*".ab,ti.

31 "attitude*".ab,ti.

32 "belief*".ab,ti.

33 "perception*".ab,ti.

34 (willingness adj2 pay).ab,ti.

35 (willingness adj2 buy).ab,ti.

36 or/22-35

37 21 and 36

Food Science and Technology Abstracts (EBSCO)

( DE "UPCYCLING" OR DE "UPCYCLED FOODS" OR DE "BY-PRODUCTS" OR DE "UPCYCLED INGREDIENTS" ) OR TI ( upcycl* N3 food* OR upcycl* N3 ingredient* OR upcycle* N3 waste* OR value-added N1 food OR value added N1 food OR added value N1 food OR circular N1 food* OR Rescue-based N1 food* OR Rescue based N1 food* OR waste-to-value N1 product* OR waste-to-value N1 food* OR food* N1 waste* ) OR AB ( upcycl* N3 food* OR upcycl* N3 ingredient* OR upcycle* N3 waste* OR value-added N1 food OR value added N1 food OR added value N1 food OR circular N1 food* OR Rescue-based N1 food* OR Rescue based N1 food* OR waste-to-value N1 product* OR waste-to-value N1 food* OR food* N1 waste* )

AND

( DE "CONSUMER ACCEPTABILITY" OR DE "CONSUMER ACCEPTANCE" OR DE "CONSUMER ATTITUDES" OR DE "CONSUMER BEHAVIOUR" OR DE "CONSUMER OPINIONS" OR DE "CONSUMER PERCEPTION" OR DE "CONSUMER PREFERENCE" OR DE "CONSUMER SATISFACTION" ) OR TI ( consumer* OR accept* OR adopt* OR prefer* OR attitude* OR belief* OR perception* OR willingness N2 pay OR willingness N2 buy ) OR AB ( consumer* OR accept* OR adopt* OR prefer* OR attitude* OR belief* OR perception* OR willingness N2 pay OR willingness N2 buy )

Agricola (EBSCOhost)

( (ZU "upcycling") or (ZU "upcycled food") or (ZU "upcycled foods") or (ZU "upcycled ingredients") ) OR TI ( ( upcycl* N3 food* OR upcycl* N3 ingredient* OR upcycle* N3 waste* OR value-added N1 food OR value added N1 food OR added value N1 food OR circular N1 food* OR Rescue-based N1 food* OR Rescue based N1 food* OR waste-to-value N1 product* OR waste-to-value N1 food* OR food* N1 waste* ) ) OR AB ( ( upcycl* N3 food* OR upcycl* N3 ingredient* OR upcycle* N3 waste* OR value-added N1 food OR value added N1 food OR added value N1 food OR circular N1 food* OR Rescue-based N1 food* OR Rescue based N1 food* OR waste-to-value N1 product* OR waste-to-value N1 food* OR food* N1 waste* ) )

AND

( (ZU "consumer acceptance") or (ZU "consumer attitudes") or (ZU "consumer preferences") or (ZU "consumer behavior") or (ZU "consumer perception") or (ZU "consumer satisfaction") ) OR TI ( consumer* OR accept* OR adopt* OR prefer* OR attitude* OR belief* OR perception* OR willingness N2 pay OR willingness N2 buy ) OR AB ( consumer* OR accept* OR adopt* OR prefer* OR attitude* OR belief* OR perception* OR willingness N2 pay OR willingness N2 buy )

Business Source Ultimate (EBSCO)

DE "FOOD industrial waste" OR TI ( upcycl* N3 food* OR upcycl* N3 ingredient* OR upcycle* N3 waste* OR value-added N1 food OR value added N1 food OR added value N1 food OR circular N1 food* OR Rescue-based N1 food* OR Rescue based N1 food* OR waste-to-value N1 product* OR waste-to-value N1 food* OR food* N1 waste* ) OR AB ( upcycl* N3 food* OR upcycl* N3 ingredient* OR upcycle* N3 waste* OR value-added N1 food OR value added N1 food OR added value N1 food OR circular N1 food* OR Rescue-based N1 food* OR Rescue based N1 food* OR waste-to-value N1 product* OR waste-to-value N1 food* OR food* N1 waste* )

AND

( DE "CONSUMER attitudes" OR DE "CONSUMER behavior" OR DE "CONSUMER preferences" OR DE "WILLINGNESS to pay" OR DE "CUSTOMER satisfaction" ) OR TI ( consumer* OR accept* OR adopt* OR prefer* OR attitude* OR belief* OR perception* OR willingness N2 pay OR willingness N2 buy ) OR AB ( consumer* OR accept* OR adopt* OR prefer* OR attitude* OR belief* OR perception* OR willingness N2 pay OR willingness N2 buy )

Web of Science Core Collection

TS = ((consumer* NEAR/3 (accept* OR adopt* OR prefer* OR attitude* OR belief* OR perception* OR intent*)) OR willingness NEAR/2 pay OR willingness NEAR/2 buy)

AND

TS=(upcycl* NEAR/3 food* OR upcycl* NEAR/3 ingredient* OR upcycle* NEAR/3 waste* OR value-added NEAR/1 food OR value added NEAR/1 food OR added value NEAR/1 food OR circular NEAR/1 food* OR Rescue-based NEAR/1 food* OR Rescue based NEAR/1 food* OR waste-to-value NEAR/1 product* OR waste-to-value NEAR/1 food* OR food* NEAR/3 "by product*" OR food* NEAR/3 byproduct* OR food* NEAR/1 waste*)

Below is a list of the databases included in the Web of Science Core Collection:

- Science Citation Index Expanded (SCI-EXPANDED)--1900-present
- Social Sciences Citation Index (SSCI)--1900-present
- Arts & Humanities Citation Index (AHCI)--1975-present
- Conference Proceedings Citation Index – Science (CPCI-S)--1990-present
- Conference Proceedings Citation Index – Social Science & Humanities (CPCI-SSH)--1990-present
- Book Citation Index – Science (BKCI-S)--2005-present
- Book Citation Index – Social Sciences & Humanities (BKCI-SSH)--2005-present
- Emerging Sources Citation Index (ESCI)--2005-present
- Current Chemical Reactions (CCR-EXPANDED)--1985-present
- Index Chemicus (IC)--1993-present
